# 

**Published:** 1893-09

**Authors:** 


The Rooms in the Medical Wing of University College, Bristol, which are devoted to library purposes, are now open to members of the Society.

---

*The following donations have been received since the publication of the list in June:*

*August* 31*st*, 1893.

| | |
|---|---|
| T. G. L'Enardi Baretti (1) | 21 volumes. |
| Alexander Carr (2) | 15 " |
| W. H. Harsant (3) | 1 volume. |
| F. P. Lansdown (4) | 1 " |
| J. E. Shaw, M.B. (5) | 2 volumes. |
| R. Shingleton Smith, M.D. (6) | 25 " |

Unbound periodicals have been received from Mr. Baretti, Mr. Lansdown, Dr. Peskett, Mr. Arthur Prichard, and Mr. A. J. Turner.

### NINTH LIST OF BOOKS.

The figures in brackets refer to the figures after the names of the donors, and show by whom the volumes were presented. The books to which no such figures are attached have either been bought from the Library Fund or received through the *Journal.*

**Acton, W.** ... ... ... *Diseases of the Urinary and Generative Organs.* (1) 2nd Ed. 1851

**Aitken, W.** ... ... *The Science and Practice of Medicine,* 2 vols. (1) 2nd Ed. 1863

**Crisfield, T.** ... ... *The Value of Hypnotism* ... ... ... ... ... ... 1893

*Dictionary, The Century.* 6 vols. ... ... ... ... ... ... ... ... [1889-91]

**Fuchs, E.** ... ... *Text-Book of Ophthalmology.* 2nd Ed. (Tr. by A. Duane) ... ... ... ... ... ... ... ... 1893

**Gorham, J.** ... ... *Tooth Extraction* ... ... ... ... ... ... ... 4th Ed. 1893

*Health, The Pathway to* ... ... ... ... ... ... ... ... ... ... ... (5) 1632

**Hill, A.** ... ... ... *The Physiologist's Note-Book* ... ... ... ... ... 1893

**Hooper, R.** ... ... *Physicians' Vade Mecum.* 8th Ed. (Revised by W. A. Guy and J. Harley) ... ... (1) 1869

*Hospitalls of K. Henry the viijth and K. Edward the vjth, The Order of the* (5) 1557

**Jewel, G.** ... ... *Midwifery* ... ... ... ... ... ... ... (1) 6th Ed. 1833

**Kelynack, T. N.** ... *Pathology of the Vermiform Appendix* ... ... ... 1893

**Liddell, J.** ... ... *The Mineral Waters of Harrogate* ... ... ... ... ... 1893

**Mueller, A.** ... ... *On Snake Poison* ... ... ... ... ... ... ... ... ... 1893

**Murrell, W.** ... ... *What to do in Cases of Poisoning* ... ... 7th Ed. 1893

*Nomenclature of Diseases, The* ... ... ... ... ... ... ... ... ... ... (1) 1869

*Pharmacopœia, British* ... ... ... ... ... ... ... ... ... ... ... ... (1) 1864

**Powell, R. D.** .. ... *On Diseases of the Lungs and Pleuræ* .. ... 4th Ed. 1893

**Skey, F. C.** ... ... *Operative Surgery* ... ... ... ... ... ... (1) 2nd Ed. 1858

**Swedenborg, E.** ... *The Brain.* Vol. II. ... ... ... ... ... ... ... (3) 1887

**Symonds, J. A.** ... *Sleep and Dreams* ... ... ... ... ... ... ... ... (1) 1851

**Thompson, Sir H.** ... *Introduction to Catalogue of Collection of Calculi of the Bladder* ... ... ... ... ... .. ... ... 1893

**Ward, S. H.** ... ... *Healthy Respiration* ... ... ... ... ... (1) 2nd Ed. 1867

**Watts, R. G.** ... ... *Consumption and its Cure* ... ... ... ... ... (1) 1868

**Williams, J. W. H.** *Unsoundness of Mind* ... ... ... ... ... ... ... (1) 1856

**Wood, G. B.** ... ... *Practice of Medicine.* 2 vols. ... ... (1) 4th Ed. 1855

" ... ... *Therapeutics and Pharmacology.* 2 vols. ... ... (1) 1856

### TRANSACTIONS, REPORTS, JOURNALS, &c.

---

| | | |
|---|---|---|
| Alienist and Neurologist, The | Vol. XIII. | 1893 |
| American Journal of Ophthalmology, The | Vol. IX. | 1892 |
| American Journal of the Medical Sciences, The | Vol. CV. | 1893 |
| American Journal of Obstetrics, The | Vol. XXVII. | 1893 |
| Archives de Neurologie | Tome XXV. | 1893 |
| Archives of Surgery | Vol. IV. | 1893 |
| Birmingham Medical Review, The | (6) Vols. X.—XXII. 1881-87; XXXIII. | 1893 |
| Brain | (6) Vol. V., 1883; (6) 1884; (6) XI.—XIV. | 1889-91 |
| Bristol Health Report, 1892 | | 1893 |
| British Gynæcological Journal, The | Vol. VIII. | 1892 |
| British Medical Journal | (4) Vol. I. for 1890; Vol. I. for | 1893 |
| Cincinnati Lancet-Clinic, The | Vol. LXIX. | 1893 |
| Dublin Journal of Medical Science, The | Vol. XCV. | 1893 |
| Edinburgh Medical Journal | Vol. XXXVIII. | 1893 |
| Glasgow Medical Journal, The | Vol. XXXIX. | 1893 |
| Hospitals— | | |
| Edinburgh Hospital Reports | Vol. I. | 1893 |
| Guy's Hospital Reports | Vol. XLIX. | 1893 |
| Saint Bartholomew's Hospital Reports | (1) Vols. III.—V. | 1867-69 |
| Lancet, The | Vol. I. for | 1893 |
| Liverpool Medico-Chirurgical Journal, The. | (6) Vols. III.—XI. | 1883-91 |
| Medical Directory, The | (2) 1874; (2) 1877-81; (1, 2) 1882; (2) | 1883-90 |
| Medical Magazine, The | Vol. I. | 1893 |
| Medical News, The | Vol. LXII. | 1893 |
| Medical Press and Circular, The | Vol. CVI. | 1893 |
| Medical Record | Vol. XLIII. | 1893 |
| Montreal Medical Journal, The | Vol. XXI. | 1893 |
| New York Medical Journal, The | Vol. LVII. | 1893 |
| Progrès Médical, Le | Tome XVII. | 1893 |
| Provincial Medical and Surgical Journal | (1) Vol. II. | 1845 |
| Retrospect of Medicine, Braithwaite's | Vol. CVII. | 1893 |
| Sei-I-Kwai Medical Journal, The | Vol. XI. | 1892 |
| Union Médicale, L' | Tome LV. | 1893 |

### PUBLICATIONS RECEIVED.

From BEAUMONT & CO., LIMITED, London:
*Hygiene.* June 23, 1893.

From BENNETT BROTHERS, LIMITED, Bristol:
*City and County of Bristol. Annual Report of the Medical Officer of Health.* 1893.

From ALFRED BOOT & SON, London:
*The British Institute of Preventive Medicine.* By M. ARMAND RUFFER, M.D.

From L. BRUCK, Sydney:
*On Snake-Poison.* By A. MUELLER, M.D. 1893.

From L. DUNCAN BUCKLEY, M.D., New York:
*Chancre of the Tonsil.* By L. DUNCAN BUCKLEY, M.D. 1893. [Reprint.]

From J. & A. CHURCHILL, London:
*Introduction to the Catalogue of the Collection of Calculi of the Bladder.* By Sir HENRY THOMPSON. 1893.

From T. CRISFIELD, London:
*The Value of Hypnotism.* By THOS. CRISFIELD. 1893.

From EVANS & WORMULL, London:
*Catalogne of Surgical Instruments.* 1893.

From CHARLES GRIFFIN & COMPANY, LIMITED, London:
*The Physiologist's Note-Book.* By ALEX. HILL, M.D. 1893.
*The Diseases of Childhood (Medical).* By H. BRYAN DONKIN, M.D. 1893.

From F. HODGSON, London:
*Temporal Welfare.* No. 1. Vol. I. June, 1893.

From GEORGE R. JESSE, Macclesfield:

*Violationism.* By GEORGE R. JESSE. Part the Second. Second Edition. 1877.

*Church Congress, Folkestone. Vivisection.* Third Edition. 1893.

*Another "Scientific" Bubble Burst.* 1893.

From H. K. LEWIS, London:

*Pathology of the Vermiform Appendix.* By T. N. KELYNACK, M.D. 1893.

*What to do in Cases of Poisoning.* By WILLIAM MURRELL, M.D. Seventh Edition. 1893.

*Text-Book of Ophthalmology.* Second Edition. By Dr. ERNEST FUCHS. Translated by A. DUANE, M.D. 1893.

*Tooth Extraction.* By JOHN GORHAM. Fourth Edition. 1893.

*Diseases of the Lungs.* By R. DOUGLAS POWELL, M.D. Fourth Edition. 1893.

From YOUNG J. PENTLAND, Edinburgh:

*Edinburgh Hospital Reports.* Volume First. 1893.

*The Mineral Waters of Harrogate.* By JOHN LIDDELL, M.D. 1893.

From F. J. REBMAN, London:

*The Provincial Medical Journal.* August, 1893.

From JOHN MORGAN RICHARDS, London:

*Medical Reprints.* July, August, 1893.

From WM. SCHEPPEGRELL, M.D., New Orleans:

*Deformities of the Nasal Septum.* By WM. SCHEPPEGRELL, M.D. [Reprint.]

From AUG. SIEGLE, London:

*The Therapist.* Vol. III. No. 8. 15th August, 1893.

From SPRAGUE & CO., London:

*On the Application of Suitable Mechanism to a Case of Amputation of Both Hands.* By F. GUSTAV ERNST. 1893.

From the ASSOCIATION FOR THE PROMOTION OF HOME AND FOREIGN TRAVEL (LIMITED), London:

*Glimpses Abroad.* 1893.

*Summer Tours and Cruises.* 1893.

From the BIRKBECK BUILDING SOCIETY, London:

*Forty-Second Annual Report.* 1893.

From the "CHRISTIAN COMMONWEALTH" PUBLISHING CO., LIMITED, London:

*The Christian Commonwealth.* July 6th, 1893.

From the CLEMENTS PRINTING WORKS, LIMITED, London:

*Talk.* Vol. I. No. 16. July 26, 1893.

From the NATIONAL TEMPERANCE LEAGUE PUBLICATION DEPÔT, London:

*The Medical Pioneer.* July, August, 1893.

From the VICTORIA STREET AND INTERNATIONAL SOCIETY FOR THE PROTECTION OF ANIMALS FROM VIVISECTION, London:

*Mr. R. T. Reid, Q.C., M.P., on Vivisection.* 1892.

*A Bird's-Eye View of a Great Question.* By SIDNEY G. TRIST. 1893.

